# Supplementary material for: Presence and Diversity of Different Enteric Viruses in Wild Norway Rats (Rattus norvegicus)
Source: Viruses. 2021 May 26;13(6):992. doi: 10.3390/v13060992 (PMC8227696; doi:10.3390/v13060992)
Supplement: Supplementary file 1 [file viruses-13-00992-s001.zip › viruses-1221140-si final.pdf]

Supplementary Table 1. Modified and designed primers and probes used for amplification and sequencing. GenBank numbers of reference sequences are as follows:

[a]HAV (DQ646426), [b]HEV (AB847308), [c]HAstV (DG415662), [d]HAstV (AB823732), [e]HAstV (FJ973620), [f]HAstV (HQ398856), [g]rAstV (KT946728), [h]rAstV (KT946727), [i]rAstV (KT946731), [k]rAstV (HM450381),

*s = sense; as = antisense

region	name *	sequence (5'–3')	position (nt)	use
HAV (VP1)	HAV5 (as)	CCATTTCAAGAGTCCACACAC	3397-3377 [a]	Screening nested RT-PCR
	HAV6 (s)	ATTCAGATTAGACTGCCTTGGTA	2817-2839 [a]	
	HAV7 (as)	CATTATTTCATGCTCCTCAGT	3300-3280 [a]	
	HAV8 (a)	GGTTTCTATTCAGATTGCAAATTA	2907-2930 [a]	
HEV (ORF1)	HEV40 (s)	CiCGCATMACMTTYTTCCARAA	3999-4020 [b]	Screening nested RT-PCR
	HEV41 (as)	GCCATGTTCCAGACDGTRTTCCA	4467-4445 [b]	
	HEV42 (s)	GCYCTGTTTGGCCCiTGGTT	4106-4125 [b]	
	HEV43 (as)	GGCTCACCiGARTGYTTCTTCCA	4431-4409 [b]	
HEV full genome	dT20AGC (as)	TTTTTTTTTTTTTTTTTTV	6945-6964 [b]	Full genome amplification
	rHEV44 (s)	GCAACCCCCGATGGAGACCATC	1-23 [b]	
	rHEV45 (s)	ATGGAGACCCATCAATATGTCGCC	11-34 [b]	
	rHEV46 (as)	TTATTCCYTCTTCCCCTCMA	4942-4923 [b]	
	rHEV47 (as)	CAGCAAAAGCACGAGCACG	5002-4984 [b]	
	rHEV48 (s)	GCCGGGCAGTCGCCAATGA	5249-5267 [b]	
	rHEV49 (s)	CATATCATGGCGACTGAGGC	5453-5472 [b]	
	rHEV50 (s)	TGGGTCTGGTGGGATGG	6644-6660 [b]	
	rHEV51 (as)	CCATCCCACCAGACCCA	6660-6644 [b]	
	rHEV52 (s)	CGTGCTCGTGCTTTTGCTG	4984-5002 [b]	
	rHEV53 (s)	CACCYAGGCCIATAATWCATGC	2259-2280 [b]	
	rHEV54 (s)	ARGAGTCCATGCAGGCYTGG	2409-2428 [b]	
	rHEV55 (as)	ATSGCCTTCTCTATGGC	4146-4130 [b]	
	rHEV56 (as)	CGGCAGTGYACAGTGGAGGA	3756-3737 [b]	
	rHEV57 (as)	ATCATGCCAYAACTTTGTCTC	3838-3818 [b]	
	rHEV58 (as)	TCATTGGCGACTGCCCGGC	5267-5249 [b]	
	rHEV59 (as)	TCAGTCGCCATGATATGCGT	5469-5450 [b]	
	rHEV60 (as)	AATCWATGGCIGGGATCTG	3059-3041 [b]	
	rHEV61 (as)	CGRTGGGACTGCAGSCGCCA	3142-3113 [b]	
	rHEV62 (s)	ACRGAGTGGCGCTGCAGTC	3107-3126 [b]	
	rHEV63 (s)	AGACAGTGYCGGGATTGGGT	1325-1344 [b]	

Supplementary Table 1. continued

region	name *	sequence (5'–3')	position (nt)	use
HEV full genome	rHEV64 (as)	GACACCCAATCCCGRCACTG	1347-1328 [b]	Full genome amplification
	rHEV65 (as)	GTGCATACCATGAGCACGCAT	562-542 [b]	
	rHEV66 (as)	CGGACACCGACGGACTTCC	2829-2811 [b]	
	rHEV67 (as)	CCCGGGACCCCCGCCAC	2805-2789 [b]	
	rHEV68 (s)	TATTGGGTGCCATTTTGTGCT	784-804 [b]	
	rHEV69 (s)	CAACAGGGGCTGCTCTTCCG	1229-1248 [b]	
	rHEV70 (s)	GGAAGAAGGAATAACACTCT	4929-4948 [b]	
	rHEV71 (s)	ATGTGTGCGGAATGCGTG	4959-4976 [b]	
	rHEV72 (as)	CGCCAGCCCTGGTTGCGGTA	5712-5693 [b]	
	rHEV73 (as)	CTTTCGCCCCGGCCACGG	4229-4212 [b]	
	rHEV74 (as)	CTGTGTGCTGTCAAACTCACT	4276-4256 [b]	
	rHEV75 (s)	ATGTGGAGGTTGTACCATCT	4337-4358 [b]	
	rHEV76 (as)	CTGTCGTGGACTGTGGCCAGA	5588-5568 [b]	
	rHEV77 (as)	GTTCATGTCCACCGACGTAG	5611-5592 [b]	
	rHEV78 (s)	AGTGTTAAGTGGGAYCAGGTC	6473-6493 [b]	
	rHEV79 (s)	CTTGAATGGTAAGGCGCTACA	6496-6516 [b]	
	rHEV80 (s)	TGGGCATCGTCTTCCGTGGC	1698-1717 [b]	
	rHEV81 (s)	CCACCCACACCGGATGAG	1787-1804 [b]	
	rHEV82 (as)	GCCACGGAAGACGATGCCCA	1717-1698 [b]	
	rHEV83 (as)	CTCATCCGGTGTGGGTGG	1804-1787 [b]	
	rHEV84 (s)	GGTTGTTGCGCGCTTGTCATC	749-769 [b]	
	rHEV85 (s)	CGTAAATCCATCCGGTCCTG	707-726 [b]	
	rHEV86 (as)	CGGATGCGACCAAGAAACAGC	208-188 [b]	
	rHEV87 (as)	CTGCAAAGGGGAGAAAAGCTC	172-152 [b]	
	rHEV88 (s)	GAGGATTTGACAGCCGACTTC	1520-1540 [b]	
	rHEV89 (s)	GCCCTTACAGATGCCCTACAC	1595-1615 [b]	
	rHEV90 (as)	ATGGCCTTCGCGGCTTGTGTG	3270-3250 [b]	
	rHEV91 (as)	TCCCCTTGACCAGAGAACACC	3231-3211 [b]	
	rHEV100 (as)	AACCAGCCTCTTACCTTTGGAG	2953-2932 [b]	
	rHEV101 (s)	GCGTTACCCACAAACTTGCC	4662-4681 [b]	
	rHEV102 (as)	GGACACCGACAGACTTCCCT	2828-2809 [b]	
	rHEV103 (s)	CCGGATGAGACATTGTACCAAAC	1796-1821 [b]	
	rHEV105 (s)	CGTCACACATCCCTCCAAAGT	1985-2005 [b]	
	rHEV106 (s)	GCCACACGTCCCTACAGAGT	1986-2005 [b]	
	rHEV107(as)	CGCGTGCAAAAGGAAAGATCAG	453-432 [b]	
	rHEV108 (as)	CATTAATAGATCGAGGGTGCGC	281-302 [b]	
	rHEV109	GATGCGACCAAGAAACAGCG	2132-2151 [b]	

Supplementary Table 1. Continued

region	name *	sequence (5'–3')	position (nt)	use
AstV (ORF1b)	AV89a (s)	CATGGGAAGCTCCTRTGCT	569-587 [c]	Screening semi-nested RT-PCR
	AV89b (s)	CATGGGAAACTTTTGTGCT	3665-3683 [d]	
	AV89c (s)	TATGGGAAGCTCCTTTGCT	4025-4043 [e]	
	AV90a (s)	TTTGGYATGTGGGTIAARCC	4021-4040 [f]	
	AV90b (s)	TTTGGRATGTGGGTIAARCC	3497-3516 [d]	
	AV90c (s)	TTTGGRATGTGGGTCAAGAG	3845-3864 [e]	
	AV91 (as)	TTTGGWCCICCCCTCCA	4364-4348 [f]	
rAstV (ORF1/2 junction)	AVr104 (as)	CTGTACCCTCGATCCTACTCG	6678-6658 [g]	Typing semi-nested RT-PCR cluster D
	AVr111 (s)	ACCCCAAYATGTCCATCGGG	4782-4801 [g]	
	AVr112a (s)	AACCTTGGGCTTGGGGATGA	5324-5343 [g]	
	AVr112b (s)	AACACTGGCCTAGGGGATGA	5324-5343 [h]	
	AVr112c (s)	AACACCGGGCTAGGTGACCA	5393-5412 [i]	
rAstV (ORF1b)	AVr108 (s)	TATGTTGTGATGCCATCTGG	1829-1848 [k]	Typing semi-nested RT-PCR cluster B
	AVr109 (as)	CCACTTGACTCTCTCATCAAC	2773-2753 [k]	
	AVr113 (as)	CAACAGCGGCTCTTGGT	2664-2648 [k]	
hAstV (ORF1b)	AV91d (as)	GGTCCKCCCCTCCAAAT	4361-4345 [f]	Typing semi-nested RT-PCR for human astrovirus
	AV91e (as)	GGTCCACCCCTCCAAAT	3831-3815 [d]	
	AV91f (as)	GGTCCKCCCCTCCAAAG	744-728 [c]	
	AV91g (as)	GGACCTCCCCTCCAAAG	4200-4184 [e]	
	AV129 (as)	AGCAWAGGAGCTTCCCAT	4043-4026 [e]	
	AV129a (as)	AGCAAAGGAGTTTCCCAT	587-570 [c]	
	AV129b (as)	AGCACAAAAGTTTCCCAT	3683-3666 [d]	
	AV130 (s)	TTYGAYTGGACRMGHTWTGATG	3431-3452 [e]	
	AV131 (s)	TTYGAYTGGACYCGMTWTGATG	3625-3646 [f]	
